# Improving the bioavailability of nintedanib by formulating inhalable ufasomes as a targeted therapy for non-small cell lung cancer

**DOI:** 10.1016/j.ijpx.2025.100482

**Published:** 2025-12-30

**Authors:** Salman M. Ghazwani, Sami Alhazmi, Salhah M. Ghazwani, Hussam M. Shubaily, Ahmed M. Wafi, Naifa Alenazi, Marwa Qadri, Amal Naif Alshammari, Wedad Mawkili, Jobran M. Moshi, Zenat Khired, Salama A. Salama

**Affiliations:** a Department of Surgery, Faculty of Medicine, Jazan University, Saudi Arabia; b Department of Pediatrics, Faculty of Medicine, Jazan University, Saudi Arabia; c Paediatric Department, King Fahad Central Hospital-Jazan, Jazan Health Cluster, Saudi Arabia; d Department of Basic Medical sciences (Pathology), Faculty of Medicine, Jazan University, Jazan, Saudi Arabia; e Basic Medical Science Department, Faculty of Medicine, Jazan University, Jazan 45142, Saudi Arabia; f Department of Pharmaceutical Science, College of Pharmacy, Princess Nourah Bint Abdulrahman University, P.O. Box 84428, Riyadh 11671, Saudi Arabia; g Department of Pharmacology and Toxicology, College of Pharmacy, Jazan University, postal code, 45142, Saudi Arabia,; h Health Research Center (HRC), Jazan University, 45142, Saudi Arabia; i Biology Department, Darb University College, Jazan University, Saudi Arabia; j Department of Pharmacology and Toxicology, College of Pharmacy, Jazan University, Jazan 45142, Saudi Arabia; k Department of Medical Laboratory Technology, College of Nursing and Health Science, Jazan University, Jazan, Saudi Arabia; l Health Research Centre, Jazan University, Jazan, Saudi Arabia; m Surgical department, faculty of medicine, Jazan University, Saudi Arabia; n Department of Biology, College of Science, Jazan University, P.O. Box. 114, Jazan 45142, Saudi Arabia

**Keywords:** Lung cancer, Lewis lung carcinoma, Targeting, Nintedanib, Bioavailability

## Abstract

The safety and effectiveness of nintedanib in treating non-small cell lung cancer (NSCLC) have been evaluated in several clinical trials. However, nintedanib exhibits low oral bioavailability due to its poor solubility and first-pass metabolism. To enhance the sustainability, targeting, bioavailability, and effectiveness of nintedanib, a targeted therapy for NSCLC was developed in the form of nebulized nintedanib ufasomes (NLU). Various NLU formulations were optimized utilizing the Design Expert software. The selected NLU was then evaluated for its aerodynamics, cytotoxicity, bioavailability, and targeting capabilities. To evaluate the effectiveness and safety of the optimal NLU formulation, a dose-dependent study was conducted using a mouse model of lung cancer induced by Lewis lung carcinoma (LLC) cell lines. The selected NLU formulation increased the sustainability, bioavailability, and targeting capability of nintedanib by 49.5 %, 6.63-fold, and 8.99-fold, respectively. Additionally, it decreased the IC_50_ value by 4.7-fold. The nebulized NLU showed better anti-tumor, anti-inflammatory, and anti-oxidative effects than oral nintedanib in terms of LDH, CEA, AFP, MDA, TNF-α, and IL-1β. The histopathological analysis confirmed these results. The safety and efficacy studies demonstrated that the nebulized NLU formulation at a dose of 100 mg/kg could serve as a viable therapy for NSCLC.

## Graphical abstract

**Figure:**
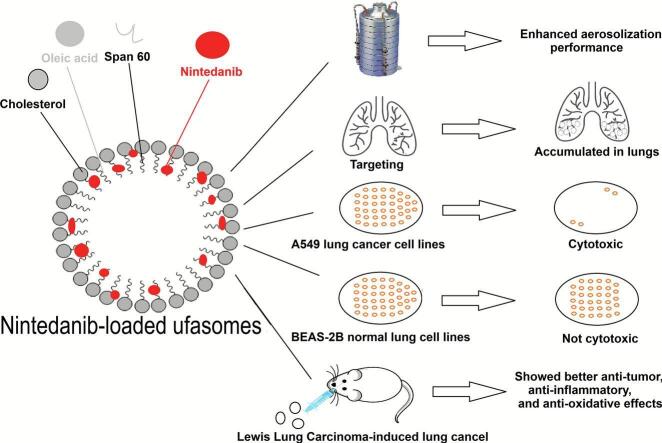

## 1 Introduction

Cancer is a disease marked by uncontrolled cell proliferation, resulting from the accumulation of mutations that disrupt normal cell growth, development, and survival (Naikoo et al., 2025). It is recognized as the second leading cause of death worldwide (Naikoo et al., 2025). On a global scale, lung cancer stands out as the most prevalent type of cancer and a major contributor to cancer-related deaths (Choudhary et al., 2025). In 2022, it was estimated that 1.82 million people would die from lung cancer, while 2.48 million new cases were diagnosed, accounting for 12.4 % of all cancer cases (Choudhary et al., 2025). Its incidence is expected to more than double by the year 2050 (Nooreldeen and Bach, 2021). Lung cancer is the leading type of cancer in terms of both incidence and mortality among men, while it ranks second among malignant tumors in women (Jiang et al., 2023). Early detection of lung cancer is essential, especially for screening high-risk populations, including smokers and individuals who are exposed to harmful fumes, oil fields, and hazardous work environments (Nooreldeen and Bach, 2021). Patients diagnosed with lung cancer may present with either small cell lung cancer (SCLC) or non-small cell lung cancer (NSCLC) (Howlader et al., 2020). More than 85 % of lung cancer cases are classified as NSCLC (Huang et al., 2022). Furthermore, only 15 % of patients diagnosed with advanced NSCLC survive for five years after their diagnosis. Consequently, NSCLC represents a significant threat to human health, as well as to economic and social development. Over the past two decades, the primary treatments for NSCLC have remained chemotherapy, radiotherapy, and surgical removal (Ettinger et al., 2021).

Nintedanib is a tyrosine kinase inhibitor that specifically targets the receptors for vascular endothelial growth factor (VEGF), fibroblast growth factor (FGF), and platelet-derived growth factor (PDGF) (Yan et al., 2023; Caglevic et al., 2015). These receptors are instrumental in promoting tumor growth and metastasis by facilitating the formation and maintenance of new blood vessels (Yan et al., 2023; Caglevic et al., 2015). As a result, nintedanib is utilized in the treatment of NSCLC. The safety and effectiveness of nintedanib in treating NSCLC have been evaluated in several clinical trials, including the VARGDO study, as well as the LUME-lung 1 and LUME-lung 2 trials (Yan et al., 2023; Ljubicic et al., 2023). However, nintedanib exhibits low oral bioavailability due to its poor intestinal absorption and limited solubility (Kala and Chinni, 2022; Zhu et al., 2020; Shukla et al., 2022). Additionally, it undergoes first-pass metabolism in the liver and is actively transported out of the intestines by P-glycoprotein transporters. This situation complicates patient compliance, as it necessitates higher doses and more frequent administration (Shukla et al., 2022). The limited options for oral administration and the dose-related side effects of nintedanib complicate its use (Shukla et al., 2022).

Nanoparticles have significantly transformed the landscape of cancer detection and treatment (Gavas et al., 2021; Ali et al., 2021). They provide several advantages that make them promising candidates for cancer therapy, including biocompatibility, reduced toxicity, enhanced stability, increased permeability and retention effects, and precise targeting (Gavas et al., 2021; Ali et al., 2021). For instance, Wang et al. developed liposomes to enhance the efficacy of paclitaxel (Wang et al., 2017), while Dianzani et al. created nanoemulsions intended for poly-chemotherapy delivery in melanoma treatment (Dianzani et al., 2020). Additionally, niosomes, liposomes, and nanostructured lipid carriers have been investigated to improve the bioavailability and therapeutic potential of nintedanib (Kala and Chinni, 2022; Zhu et al., 2020; Shukla et al., 2022). To date, no studies have established a targeted therapy for NSCLC through the inhalation of nintedanib ufasomes. Ufasome nanocarriers are made from a non-ionic surfactant (NIS), cholesterol, and free fatty acids (FFA) (Abd-Elal et al., 2016; Gulshan et al., 2023; Illastria Rosalina et al., 2023). Ongoing research is exploring ufasomes as a potential alternative to liposomes, primarily because they lack phospholipids, which are chemically unstable and susceptible to degradation from oxygen and water. Ufasomes also outperform niosomes by incorporating FFA, which enhances drug stability and absorption rates (Abd-Elal et al., 2016; Gulshan et al., 2023). They improve medication sustainability, targeting, bioavailability, and efficacy (Abd-Elal et al., 2016; Gulshan et al., 2023; Illastria Rosalina et al., 2023). Furthermore, ufasomes represent a novel type of nano-cargo designed to enhance the anticancer properties of drugs such as curcumin and quercetin (El Taweel et al., 2023; Aboud et al., 2022).

An emerging area of focus is the targeted and localized delivery of chemotherapeutics to the lungs using nanoparticle carriers, addressing the challenges posed by conventional chemotherapy. Systemic chemotherapy often leads to the delivery of anticancer medications to unintended areas, which can diminish treatment effectiveness and cause serious systemic side effects (Ahmad et al., 2015). The inhalation route improves drug localization in the lungs, thereby enhancing efficacy, reducing side effects, and facilitating a rapid drug response (Shukla et al., 2022; Saafan et al., 2021; Wang et al., 2024). Currently, nebulizer therapy shows promise as a treatment option for lung cancer (Feng et al., 2023; Al Khatib et al., 2023). Advantages of using a nebulizer for inhalation therapy include the ease of administering higher doses, the accelerated onset of action, and reduced mess (Jayeshkumar et al., 2019).

No studies have yet demonstrated a targeted therapy for NSCLC via the inhalation of nintedanib ufasomes (NLU). No research has examined the improvement of nintedanib's efficacy and bioavailability via the formulation of a nebulized ufasome. To enhance the sustainability, targeting, bioavailability, and effectiveness of nintedanib, a targeted therapy for NSCLC was developed in the form of a nebulized nintedanib ufasome (NLU). Various formulations of NLU were optimized utilizing the Box-Behnken design. The optimal NLU formulation was then evaluated for its aerodynamics, cytotoxicity, bioavailability, and targeting capabilities. To evaluate the effectiveness and safety of the selected NLU formulation, an in vivo study was conducted using a mouse model of lung cancer induced by Lewis lung carcinoma (LLC) cell lines.

## 2 Materials and methods

### 2.1 Materials

Agitech Pharmaceutical Company (Cairo, Egypt) was the source of Span 60, methanol, chloroform, oleic acid, and cholesterol.

### 2.2 Fabrication and optimization of nintedanib ufasomes

The independent variables included cholesterol, FFA, and NIS. Vesicle size (VS) and encapsulation efficiency (EE%) were designated as the dependent variables. A Box-Behnken design, using Design Expert software (Minneapolis, USA), was employed to assess the impact of cholesterol (X_1_), Span 60 (X_2_), and oleic acid (X_3_) on both EE% and VS. Table 1 presents the fifteen unique NLU formulations created using this method.

**Table 1:** Box-Behnken design of nintedanib ufasome formulations.

Formulations	Cholesterol amount (mg)	Span 60 amount (mg)	Oleic acid amount (mg)	Encapsulation Efficiency (%)	Vesicle size (nm)
F1	40	65	10	87.06 ± 0.61	270.0 ± 4.26
F2	40	65	50	91.61 ± 0.52	288.1 ± 5.28
F3	20	50	10	85.04 ± 0.49	260.6 ± 5.89
F4	20	65	30	93.42 ± 0.51	270.2 ± 4.64
F5	60	50	50	91.16 ± 0.44	305.4 ± 5.85
F6	60	50	10	88.19 ± 0.48	270.9 ± 3.99
F7	40	35	10	82.19 ± 0.50	259.3 ± 4.23
F8	60	65	30	95.29 ± 0.58	280.7 ± 5.07
F9	60	35	30	90.58 ± 0.52	270.2 ± 4.66
F10	40	50	30	80.41 ± 0.53	249.5 ± 5.04
F11	20	50	50	90.19 ± 0.47	299.1 ± 3.87
F12	20	35	30	88.16 ± 0.52	260.8 ± 5.25
F13	40	50	30	80.18 ± 0.53	249.0 ± 5.35
F14	40	35	50	87.29 ± 0.46	289.1 ± 5.42
F15	40	50	30	80.41 ± 0.59	240.9 ± 4.10

Data are displayed as mean ± standard deviation (SD) (n = 3).

The analysis of variance (ANOVA) was employed to assess the results of the VS and EE% metrics. The best-fitting model for the data was determined using several criteria, including lack of fit, F-value, adequate precision, *p*-value, and the coefficient of determination (R^2^) (Sayed et al., 2020). To verify the selected model for EE% and VS, normal probability plots were generated (Raghupathy and Amirthagadeswaran, 2014). To explore the impact of X_1_, X_2_, and X_3_ on both EE% and VS, regression equations and 3D plots were generated (Raghupathy and Amirthagadeswaran, 2014).

By utilizing the desirability index (DI) during the optimization process, we identified the optimal levels of cholesterol, Span60, and oleic acid to maximize the EE% while minimizing the VS (Albash et al., 2022). The program selects the optimal formulation based on the highest DI value. We validated these findings by comparing the selected formulation's VS and EE% with the program's predicted data.

### 2.3 Formation of NLUs

NLUs were synthesized using the thin-film hydration technique (Tawfik et al., 2021). Initially, a round-bottom flask was filled with a 15 ml mixture of chloroform and methanol (2:1, *v*/v), which contained nintedanib (10 mg), Span 60, oleic acid, and cholesterol. A thin, dry film was formed on the walls of the round-bottom flask by gradually evaporating the organic solvent under reduced pressure using a rotary evaporator (Heidolph VV 2000, Burladingen, Germany) set at 40 °C and 100 rpm. The resulting film was then hydrated with 10 ml of phosphate-buffered saline at a pH of 7.4 for two hours at 60 °C and 60 rpm. The resulting nanodispersion was stored at a temperature of 4 °C.

### 2.4 In vitro evaluation of the NLU formulation

#### 2.4.1 Determination of encapsulation efficiency (EE%)

The freshly prepared nano-dispersion underwent centrifugation at 20,000 rpm for 1 h at 4 °C using a cooling ultracentrifuge (Sigma 3–30 KS, Osterode, Germany) to separate the unentrapped drug. After removing the residue, the unentrapped drug was recentrifuged at 4 °C and 20,000 rpm for an additional hour. Spectrophotometric measurements of the resulting supernatant were performed using a Shimadzu UV1650 (Kyoto, Japan) at a wavelength of 389 nm, corresponding to the λ_max_. This method proved effective for quantifying nintedanib concentrations, as the linearity of the regression equation for nintedanib in the range of 0.2 to 1 μg/ml yielded an R^2^-value of 0.9997. Each experiment was performed in triplicate. The results are presented as the mean ± standard deviation (SD) after calculating the EE% as follows (El-Salamouni et al., 2021):

(1) $$\mathrm{EE}\;\left(\%\right)=\frac{\text{Total amount of}\;\mathrm{nintedanib}-\mathrm{free nintedanib}}{\text{Total amount of}\;\mathrm{nintedanib}}$$

### 2.5 Vesicle size (VS), zeta potential (ZP) and polydispersity index (PDI) analysis

The Malvern Zetasizer Nano (Malvern, Worcestershire, UK) was employed to assess the VS, ZP, and PDI (Tawfik et al., 2021). Before measurement, one ml of the NLU suspension was diluted with nine ml of distilled water. Each experiment was conducted in triplicate, and the results were reported as the mean ± SD.

#### 2.5.1 Differential scanning calorimetry (DSC)

DSC (Shimadzu, Germany) was employed to investigate the crystalline structure of nintedanib and the thermal behavior of NLU, cholesterol, oleic acid, and Span 60 from 25 to 350 °C at a rate of 10 °C/min (Abou-Taleb et al., 2024).

#### 2.5.2 Fourier transform infrared spectroscopy (FTIR)

FTIR (Bruker, Alpha, Germany) was employed to investigate the chemical interactions in the NLU formulation, which includes nintedanib, oleic acid, Span 60, and cholesterol at 400 to 4000 cm^−1^ (Fouad et al., 2024).

### 2.6 Morphologic examination

A scanning electron microscope (SEM, Zeiss DSM 982 Gemini, LEO Oberkochen, Germany) (Sayed et al., 2020) and a transmission electron microscope (TEM, JEOL JEM1230, Tokyo, Japan) (Abou-Taleb et al., 2023) were utilized to examine the morphology of the optimal NLU formulation. The TEM analysis commenced with a one-minute adhesion period for a drop of NLU dispersion placed on a carbon-coated copper grid. Additionally, a piece of filter paper was employed to absorb any excess dispersion. Prior to analysis in the SEM apparatus, a gold coating was applied to the formulation.

### 2.7 Release kinetics

The solubility of nintedanib was measured spectrophotometrically three times in a phosphate buffer (pH 7.4) that contained 0.5 % Tween 80 (PT80) at a λ_max_ of 389 nm (Abouelmagd et al., 2015).

To investigate the drug release from an optimal NLU and nintedanib suspension, we employed the dialysis membrane technique (Fouad et al., 2024). Each glass cylinder was sealed with a dialysis membrane that contained either a sample of optimal NLU (equivalent to 3 mg of nintedanib) or a free nintedanib suspension. Next, we added 50 ml of PT80 (pH 7.4) to beakers and lowered the glass cylinders into them. The cylinders were configured to rotate continuously at 100 rpm within a temperature range of 37 ± 0.5 °C. To maintain constant sink conditions, we took 3 ml samples at intervals of 0.5, 1, 2, 3, 4, 5, 6, and 8 h, replacing the removed volume with fresh medium. We subsequently conducted spectrophotometric measurements at a λmax of 389 nm, and the results were reported as the mean ± SD.

The DDSolver program was employed to determine the release kinetics for optimal NLU and nintedanib suspension (Kirimlioğlu and Öztürk, 2020). Rankings based on the Akaike information criterion (AIC), model selection criterion (MSC), and R^2^ values were utilized to identify the best-fitting model. To analyze the release kinetic mechanism, the release exponent (n) from the Korsmeyer-Peppas model was calculated. Furthermore, to assess the sustained release effect of the optimal NLU formulation, the mean dissolution time (MDT T50) was computed (Kirimlioğlu and Öztürk, 2020).

### 2.8 Stability study

The physical stability of the optimal NLU was assessed by measuring the EE% and VS. Glass vials containing samples of the optimal NLU formulation were stored at three different temperatures—4 °C, 25 °C, and 40 °C—over a period of three months. Samples from each formulation were collected monthly to evaluate their EE% and VS.

### 2.9 Aerodynamic characterization

One established method for assessing the particle size distribution in the respiratory system is the Anderson Cascade Impactor (ACI) (Nahar et al., 2013). In this study, we administered 200 μg of nintedanib suspension and the optimized NLU. Five doses were actuated throughout the experiment. The jet nebulizer was used to deliver the optimized NLU (Abdelrahim, 2011). The ACI setup, which included a vacuum pump, maintained a flow rate of 28.3 L/min. The amount of drug deposited on each stage was quantified using the liquid chromatography–mass spectrometry method (Xu et al., 2015). The C18 column from Zorbax, with a particle size of 3.5 μm and dimensions of 4.6 × 50 mm, was employed for the analysis. A gradient elution technique was utilized at a flow rate of 0.4 ml/min, with the mobile phase consisting of acetonitrile and 0.1 % formic acid. The results demonstrated that the LC-MS/MS method showed linearity within the range of 0.1 to 500 ng/ml, achieving R^2^ values of 0.999. The limit of quantification was established at 0.1 ng/ml. Important metrics that can be assessed using data collected from Copley inhalers include geometric standard deviation (GSD), mass median aerodynamic diameter (MMAD), fine particle dose (FPD), and fine particle fraction percent (FPF).

#### 2.9.1 Cytotoxicity assay

The cytotoxicity assay was conducted at the National Cancer Institute in Egypt, using A549 NSCLC cell lines and BEAS-2B, a human bronchial epithelial cell line. A549 cells (5.9 × 10^6^/ml) and BEAS-2B cells (6.1 × 10^5^/ml) were seeded in 96-well plates and incubated in a humidified environment with 5 % CO₂ at 37 °C for 24 h (Gi et al., 2019). Following the incubation, various concentrations of 0.001, 0.01, 0.1, 1, and 10 μg/ml of the optimized NLU and nintedanib were added to A549 cells. The cells were then incubated for an additional 48 h at 37 °C in a CO₂ environment. BEAS-2B cells received treatment with optimal NLU at the same concentrations and were also incubated for 48 h under identical conditions. The MTT colorimetric assay was employed to assess cell viability and proliferation. After incubation with nintedanib and optimal NLU, each well was supplemented with 50 μl of MTT solution. The plates were then placed in an incubator set at 37 °C with 5 % CO₂ for four hours. To dissolve the crystals, 100 μl of DMSO was added to each well. Once the formazan product was fully dissolved in DMSO, its absorbance was measured at 550 nm, revealing a dark blue color. Cell viability and the drug concentration that inhibits cell lines by 50 % (IC50) were determined.

### 2.10 In vivo study

#### 2.10.1 Study protocol

The animal approval was conducted in accordance with the ethical standards outlined by the ARRIVE guidelines. The Institutional Animal Ethics Committee (IACUC024–013) reviewed and approved the animal studies associated with this research.

### 2.11 Pharmacokinetic study

The twelve adult male Wistar rats, each weighing between 200 and 250 g, were housed in clean polypropylene cages in a standard air-conditioned animal facility. The environmental conditions consisted of a temperature maintained at 22 ± 2 °C, a relative humidity ranging from 50 % to 70 %, and a 12-h light/dark cycle. The statistical significance of the sample size for the animal study was evaluated using G*Power software (version 3.1.9.2). After one week of acclimatization, the rats were randomly assigned to two groups: one group received nebulized NLU (10 mg/kg), while the other group received oral nintedanib (10 mg/kg). Each group consisted of six animals.

Blood samples were obtained from the retroorbital plexus and centrifuged at 3500 rpm for 15 min to isolate the serum. Two ml of acetonitrile were added to the serum using a vortex mixer, followed by centrifugation at 10,000 rpm for 10 min. The mobile phase was introduced, and the concentration of nintedanib was quantified using chromatography (Xu et al., 2015). The PK Solver program was utilized to assess pharmacokinetic parameters such as maximal plasma concentration (C_max_), half-life (t_0.5_), and relative bioavailability (Prusty et al., 2022).

### 2.12 Biodistribution study

Gold's high X-ray attenuation value made it suitable for use as a cap for optimized NLU and nintedanib (Salem et al., 2018). To synthesize gold nanoparticles, a solution of gold (0.001 M) was prepared, and 0.04 M trisodium citrate was added to it. The mixture was stirred for 5 min until an initial color change was observed (Hamzawy et al., 2017). The optimal NLU was centrifuged, and the resulting pellets were combined with the gold solution. This mixture underwent ultrasonication for one hour to produce the optimal NLU gold nanoparticles. The nintedanib crude drug was mixed with the gold solution. This mixture was subjected to ultrasonication for one hour to create the free nintedanib gold nanoparticles. The oral nintedanib group received free nintedanib gold nanoparticles, while the nebulized NLU group was administered optimized NLU gold nanoparticles. A computed tomography scanner (Sensation 64, Siemens CO, Erlangen, Germany) was employed to visualize the distribution of nintedanib (Salem et al., 2018).

#### 2.12.1 Induction of lung cancer and treatment protocol

The thirty-six male BALB/c mice, each weighing between 25 and 30 g, were housed in clean polypropylene cages within a standard air-conditioned animal facility. The environmental conditions were carefully controlled, maintaining a temperature of 22 ± 2 °C, a relative humidity between 50 % and 70 %, and a 12-h light/dark cycle. The sample size's statistical significance for the animal study was assessed using G*Power software (version 3.1.9.2). Following a one-week acclimatization period, the mice were randomly divided into six groups: a negative control group, a disease group, an oral nintedanib group, a nebulized NLU50 group, a nebulized NLU100 group, and a nebulized NLU200 group. Each group is comprised of six animals.

The induction of lung cancer was achieved using Lewis lung cancer (LLC) cell lines in the disease group, along with the groups that received treatment with oral nintedanib, nebulized NLU50, nebulized NLU100, and nebulized NLU200 (Tanaka et al., 2023). Each mouse was anesthetized, and a surgical incision was made around the neck. A suspension containing 1 × 10^6^ LLC cells was then injected directly into the trachea (Tanaka et al., 2023).

The study's negative control group maintained a standard diet throughout the experiment. The disease control group received no treatment. The oral nintedanib group was given a free nintedanib suspension at a dosage of 200 mg/kg for 28 days. The nebulized NLU50 group received NLU at a dosage of 50 mg/kg for 28 days. The nebulized NLU100 group received NLU at a dosage of 100 mg/kg for 28 days. The nebulized NLU200 group received NLU at a dosage of 200 mg/kg for 28 days.

#### 2.12.2 Assessment of anti-tumor activity

The blood samples were obtained from the tail vein of mice in all groups. Following collection, the blood was centrifuged at 3000 rpm for 15 min, and the resulting serum was used to assess the levels of carcinoembryonic antigen (CEA), alpha-fetoprotein (AFP), and lactate dehydrogenase (LDH) with the aid of appropriate analytical kits (Hamzawy et al., 2017).

Lungs were homogenized for 30 min. The homogenized samples were then centrifuged at 5000 rpm for 20 min. Subsequently, the supernatants were analyzed to assess malondialdehyde (MDA), glutathione (GSH), tumor necrosis factor-alpha (TNF-α), and interleukin-1 beta (IL-1β) levels (Hamzawy et al., 2017).

### 2.13 Safety study

Blood samples were collected from mice in the following groups: the control negative group, the oral nintedanib group, the nebulized NLU50 group, the nebulized NLU100 group, and the nebulized NLU200 group. After collection, the blood was centrifuged at 3000 rpm for 15 min, and the resulting serum was used to evaluate the levels of alanine aminotransferase (ALT), aspartate aminotransferase (AST), platelet counts, white blood cells (WBCs), neutrophils, lymphocytes, and monocytes (Ayen and Kumar, 2012).

#### 2.13.1 Histopathological analysis

Lung tissue was fixed using 10 % formalin and embedded in paraffin wax. The sections were stained with hematoxylin and eosin for histological examination (Akhouri et al., 2020).

### 2.14 Statistical analysis

Data are presented as means with standard deviations (mean ± S.D.). All statistical analyses were performed using SPSS software (SPSS Inc., Chicago, IL, USA). The significance level for all statistical analyses was determined using Tukey's post hoc test.

## 3 Results

### 3.1 Design and optimization of NLU

According to the pre-formulation study, Span 60 was chosen as a NIS, oleic acid as an FFA, and cholesterol as an independent variable for our research.

Table 1 presents the composition of each NLU formulation created using Design Expert software, based on the Box-Behnken design. It also includes the results for the EE% and the VS for all NLU formulations.

Table 2 presents various criteria utilized by the Box-Behnken design to identify the model for EE% and VS. The quadratic model was identified as the most suitable for both EE% and VS.

**Table 2:** Criteria for selecting models that best fit the responses of nintedanib ufasomes.

Parameter	Models					
	Encapsulation efficiency			Vesicle size		
	Linear	2FI	Quadratic	Linear	2FI	Quadratic
*p-*value	0.0031	0.9612	<0.0001	0.0012	0.9181	<0.0001
F-value	5.04	0.0212	1089.28	7.02	0.0211	398.56
R^2^	0.3568	0.4265	0.9984	0.4635	0.4268	0.9986
Lack of Fit	<0.0001	<0.0001	0.7805	<0.0001	<0.0001	0.5687
Adjusted R^2^	0.3025	0.4036	0.9983	0.4265	0.4025	0.9923
Predicted R^2^	0.3125	0.3986	0.9922	0.3986	0.3987	0.9912
Adequate precision	7.0236	4.6987	66.8564	8.1258	5.0256	49.0256

**Fig. 1:**
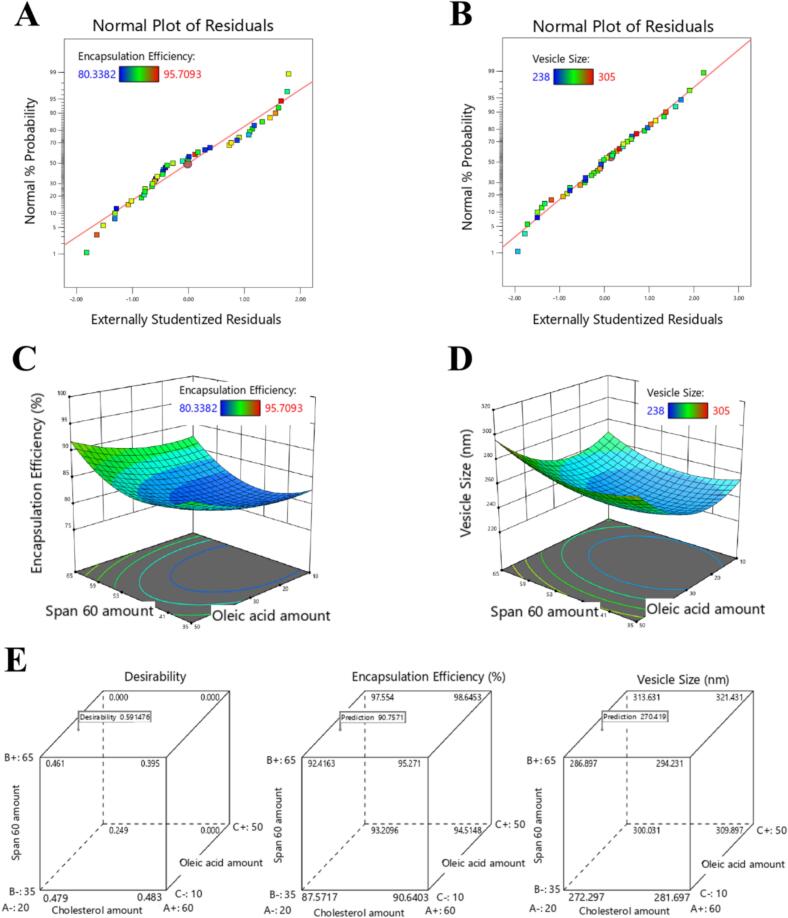
A) Normal probability plot of residuals for encapsulation efficiency of nintedanib ufasomes; B) Normal probability plot of residuals for vesicle size of nintedanib ufasomes; C) 3D graph plot illustrates how independent variables affect the encapsulation efficiency of nintedanib ufasomes; D) 3D graph plot illustrates how independent variables affect the vesicle size of nintedanib ufasomes; E) Cube plot illustrates the desirability and the composition of the optimal formulation for nintedanib ufasomes.

Fig. 1A and B display the normal probability plots for both EE% and VS, respectively. The results confirm that the quadratic model is the most suitable for both EE% and VS.

Fig. 1C and D present three-dimensional plots illustrating the impact of independent factors on EE% and VS. Cholesterol, oleic acid, and Span 60 positively (*p*-value <0.0001) influenced both EE% and VS. The NLU6 formulation, which contained higher levels of cholesterol, demonstrated EE% and VS values that exceeded those of the NLU3 formulation, which contained lower levels of cholesterol, by 3.81 % and 5.03 %, respectively. The NLU1 formulation, which contained higher levels of Span 60, demonstrated EE% and VS values that exceeded those of the NLU7 formulation, which contained lower levels of Span 60. The differences in EE% and VS were 5.29 % and 6.59 %, respectively. The NLU2 formulation, which had higher levels of oleic acid, achieved EE% and VS values that exceeded those of the NLU1 formulation, which contained lower levels of oleic acid, by 5.45 % and 7.07 %, respectively.

(2) $$\mathrm{EE}\;\left(\%\right)=+81.22+1.22\times {\;}_{1}+2.16\times {\;}_{2}+2.07\times {\;}_{3}–0.0602X_{1}X_{2}–0.4063X_{1}X_{3}–0.1156X_{2}X_{3}+6.65\times {{}_{1}}^{2}+4.12\times {{}_{2}}^{2}+1.68\times {{}_{3}}^{2}$$

(3) $$\mathrm{VS}\;\left(\mathrm{nm}\right)=+245.7+4.26\times {\;}_{1}+6.64\times {\;}_{2}+13.68\times {\;}_{3}–0.5063X_{1}X_{2}–0.1246X_{1}X_{3}–0.2489X_{2}X_{3}+21.09\times {{}_{1}}^{2}+12.36\times {{}_{2}}^{2}+21.46\times {{}_{3}}^{2}$$

Fig. 1E presents the DI alongside the composition of the optimal formulation for the NLU. The chosen formulation consists of cholesterol (25 mg), Span 60 (60 mg), and oleic acid (25 mg). This specific combination was identified as the optimized NLU, as it achieved the highest DI of 0.609. Furthermore, the optimized NLU demonstrated an EE% of 91.02 ± 0.64 % and a VS of 270.5 ± 5.24 nm.

### 3.2 In vitro characterization

#### 3.2.1 PDI and ZP analysis

**Fig. 2:**
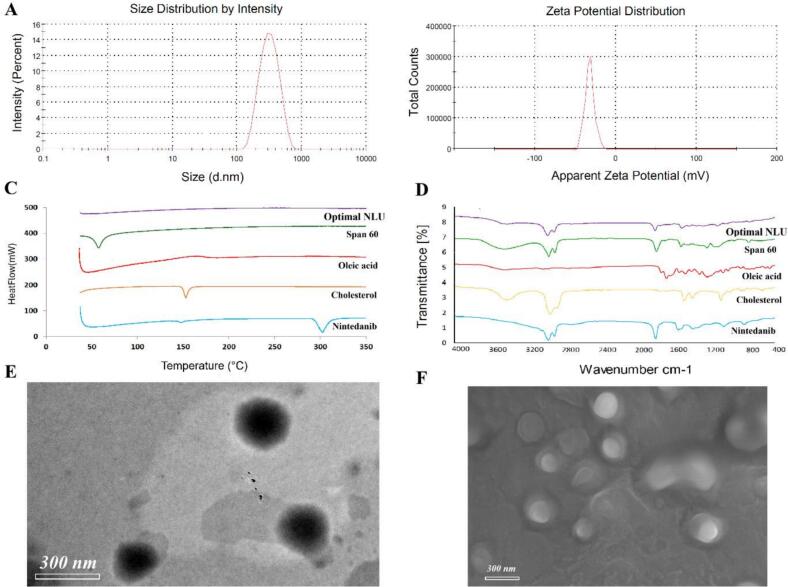
A) Vesicle size distribution of nintedanib ufasomes; B) Zeta potential distribution of nintedanib ufasomes; C) Differential scanning calorimetry of nintedanib ufasomes; D) Fourier transform infrared spectroscopy of nintedanib ufasomes; E) Transmission electron microscopy of nintedanib ufasomes; F) Scanning electron microscopy of nintedanib ufasomes.

Fig. 2A shows the vesicle size distribution of the optimized NLU, revealing a uniform distribution with a PDI of 0.206 ± 0.02. Meanwhile, Fig. 2B depicts the ZP of the optimized NLU vesicles, demonstrating that the vesicles have a negative charge of −34.1 ± 0.64 mV.

#### 3.2.2 DSC analysis

Fig. 2C illustrates the DSC scan for optimized NLU, nintedanib, cholesterol, Span 60, and oleic acid. The DSC scan of nintedanib displayed two endothermic peaks at 144 °C and 309 °C. The DSC scan for Span 60 showed an endothermic peak at 58.35 °C, while cholesterol exhibited a peak at 150.59 °C, and oleic acid displayed a peak at 167.04 °C. Notably, the DSC scan of the optimized NLU showed no peaks.

#### 3.2.3 FTIR analysis

Fig. 2D presents the FTIR spectra for optimized NLU, nintedanib, cholesterol, Span 60, and oleic. Bands at 1457 cm^−1^, 1712 cm^−1^, 1422, and 1288 cm^−1^ represent the amide, ester, and C—N groups of nintedanib. Bands at 2944.25 cm^−1^ and 2834.86 cm^−1^ indicate -CH₂ of oleic acid. Bands at 3408.14 cm^−1^, 2920.22 cm^−1^, and 1466.27 cm^−1^ represent the O—H, aliphatic C—H, and -CH₃ of Span 60. For cholesterol, bands at 3404.52 cm^−1^, 2935.84 cm^−1^, 1659.31 cm^−1^, and 1460.97 cm^−1^, represent the O—H, CH₂, C=C, and CH₂. A striking similarity to the bands of nintedanib and other components was shown in the FTIR of the optimized NLU.

#### 3.2.4 Morphology analysis

Fig. 2E and F illustrate the TEM and SEM of the optimized NLU. It appears that the optimized NLU consists of non-aggregating, uniformly sized spherical nanovesicles.

### 3.3 Release kinetics

Based on the results of the equilibrium solubility study, a 50 ml release medium consisting of PT80 was selected to maintain sink conditions.

**Fig. 3:**
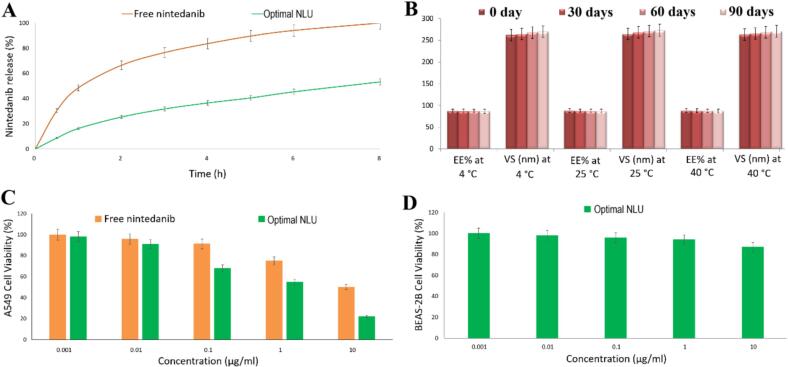
A) Release profile of nintedanib ufasomes; B) Stability profile of nintedanib ufasomes; C) Cytotoxicity of nintedanib ufasomes against A549 lung cancer cell lines; D) Safety of nintedanib ufasomes against BEAS-2B normal lung cells. The results were represented as mean ± SD (*n* = 3).

The release of the optimized NLU is compared to that of the crude nintedanib drug, as shown in Fig. 3A. The crude nintedanib drug released 99.02 ± 0.61 % of its content, whereas the optimized NLU released 49.99 ± 0.71 %. There was a 49.50 % reduction (student's *t*-test, *p*-value <0.0001) in drug release when comparing the optimized NLU to the crude nintedanib drug.

Table 3 presents the various criteria employed by the DDsolver to identify the optimal kinetic model for NLU and nintedanib. The analysis indicates that the Korsmeyer-Peppas model is the most suitable for both the optimized NLU and nintedanib, with “n” values of 0.307 and 0.352, respectively. These values indicate that both formulations exhibit a Fickian diffusion mechanism. The MDT (T50) for the optimal NLU formulation was 20.98 ± 1.68 h, compared to 7.24 ± 0.64 h for free nintedanib. The optimal NLU significantly (student's *t*-test, *p*-value <0.0001) increased the MDT of free nintedanib by 2.90-fold.

**Table 3:** Release kinetics of optimum nintedanib ufasomes.

Parameter	Release Kinetic Models					
	Free nintedanib			Optimum nintedanib ufasomes		
	R^2^	MSC	AIC	R^2^	MSC	AIC
Zero order	0.4465	0.3063	79.5685	0.8025	1.4256	52.0256
First order	0.9812	2.9525	47.6956	0.9468	2.2463	44.2056
Higuchi	0.9502	2.1263	56.0869	0.9688	3.9968	29.8865
Korsmeyer-Peppas	0.9981	3.4563	45.2956	0.9972	4.8965	22.4236
Hixson-Crowell	0.9656	1.8236	56.6986	0.9102	1.9002	47.6897

Coefficient of determination (R^2^), Akaike information criterion (AIC), Model selection criterion (MSC). The results were represented as mean ± SD (n = 3).

### 3.4 Stability studies

Fig. 3B presents the results of the stability study for the optimal NLU formulation. Following a three-month storage period at 4, 25, and 40 °C, no significant changes in EE% or VS (ANOVA, p-value >0.05) were observed.

### 3.5 Aerodynamic characterization

The optimized NLU had an FPD of 138.52 ± 7.56 μg and an FPF of 86.02 ± 3.86 %. In comparison, the values for free nintedanib were 70.62 ± 3.97 μg and 45.62 ± 4.12 %. The optimized NLU significantly enhanced the free nintedanib's FPD by a factor of 1.96 and its FPF by a factor of 1.89, as determined by a student's *t*-test (*p*-value <0.0001). The optimized NLU had a MMAD of 2.95 ± 0.29 μm and a GSD of 1.42 ± 0.35. In comparison, free nintedanib had values of 4.56 ± 0.34 μm for MMAD and 3.24 ± 0.26 for GSD. The optimized NLU significantly (student's *t*-test, *p*-value <0.0001) decreased the MMAD and GSD of free nintedanib. by 35.31 % and 56.17 %, respectively.

### 3.6 Cytotoxicity study

Fig. 3C presents the results of the cytotoxicity study conducted on A549 cells, comparing the optimized NLU formulation and free nintedanib. The results show that cell viability declines as the concentration of either the optimal NLU formulation or free nintedanib increases. The optimized NLU exhibited an IC50 of 2.1 μg/ml, whereas the nintedanib showed an IC50 of 9.9 μg/ml. This suggests that the IC50 for the optimized NLU is 4.7 times lower (student's t-test, p-value <0.0001) compared to nintedanib.

Fig. 3D illustrates the safety profile of the optimized NLU in BEAS-2B normal cells. The results indicated that the optimum NLU formulation maintained over 85 % cell viability after a 48-h incubation, with an IC50 value of 47.8 μM.

### 3.7 In vivo study

#### 3.7.1 Pharmacokinetic study

**Fig. 4:**
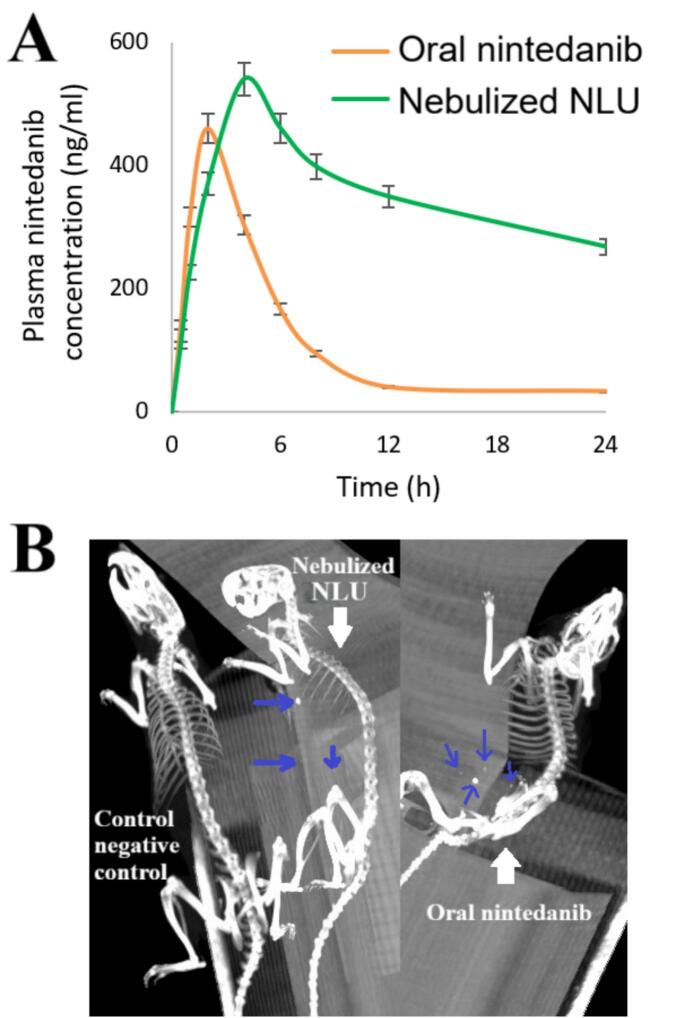
A) Pharmacokinetic profile of nintedanib ufasomes; B) Tissue distribution of nintedanib ufasomes using computed tomography imaging. Blue arrow: indicate the distribution of nintedanib. The results were represented as mean ± SD (*n* = 6). (For interpretation of the references to color in this figure legend, the reader is referred to the web version of this article.)

Fig. 4A presents the plasma concentration over time for nebulized NLU in comparison to oral nintedanib. The nebulized NLU achieved a Cmax of 559.64 ± 36.26 ng/ml and an AUC0-inf of 19,634.27 ± 264.34 ng·h/ml. In contrast, oral nintedanib exhibited values of 461.02 ± 29.24 ng/ml for Cmax and 2963.39 ± 126.32 ng·h/ml for AUC0-inf. These findings indicate that the Cmax and AUC0-inf for the optimized NLU are 1.22 and 6.63 times higher, respectively (student's t-test, *p*-value <0.0001), compared to nintedanib.

The Tmax, t_0.5_, and MRT values for the nebulized NLU were measured at 4 h, 29.65 ± 1.23 h, and 43.09 ± 1.31 h, respectively. In contrast, the corresponding values for oral nintedanib were 2 h, 6.03 ± 0.87 h, and 8.28 ± 0.96 h. The nebulized NLU significantly (student's t-test, *p*-value <0.0001) enhanced the Tmax, t0.5, and MRT of oral nintedanib, with increases of 2-fold, 4.92-fold, and 5.20-fold, respectively.

#### 3.7.2 CT imaging

Fig. 4B illustrates the distribution of the drug across various organs following the administration of nebulized NLU compared to oral free nintedanib. In the nebulized NLU group, the majority of the drug accumulated in the lungs (indicated by the blue arrow), with smaller amounts found in the liver, spleen, and kidneys. Conversely, in the case of oral nintedanib, most of the drug was distributed in the liver, bladder, and kidneys, while a minor portion was found in the lungs.

#### 3.7.3 Assessment of anti-tumor activity

**Fig. 5:**
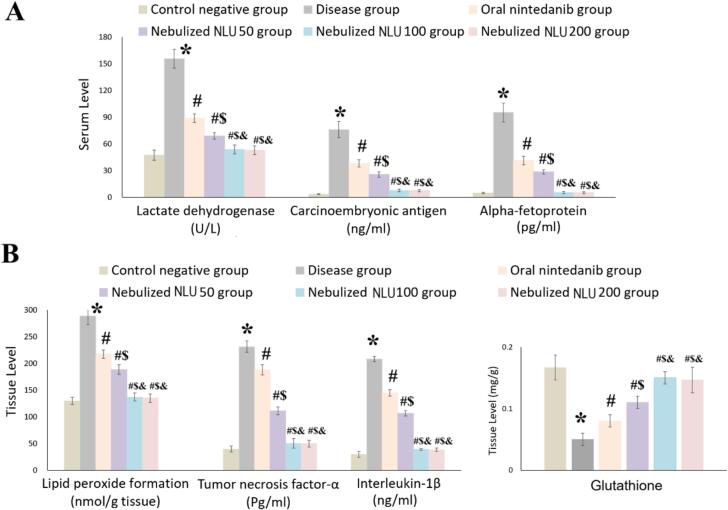
A) Effect of administration of oral nintedanib, nebulized nintedanib ufasomes at a dose of 50 mg/kg (NLU50), nebulized nintedanib ufasomes at a dose of 100 mg/kg (NLU100), and nebulized nintedanib ufasomes at a dose of 200 mg/kg (NLU200) on serum levels of tumor biomarkers; B) Effect of administration of oral nintedanib, nebulized nintedanib ufasomes at a dose of 50 mg/kg (NLU50), nebulized nintedanib ufasomes at a dose of 100 mg/kg (NLU100), and nebulized nintedanib ufasomes at a dose of 200 mg/kg (NLU200) on tissue levels of lipid peroxidation and inflammatory cytokines. * indicates a significant difference compared to control negative group at p-value <0.05; # indicates a significant difference compared to disease group at *p*-value <0.05; $ indicates a significant difference compared to oral nintedanib group at p-value <0.05; & indicates a significant difference compared to nebulized NLU50 group at p-value <0.05. The results were represented as mean ± SD (*n* = 6).

Fig. 5 illustrates how various treatments impact the levels of LDH, CEA, AFP, MDA, TNF-α, IL-1β, and GSH.

The disease group demonstrated a significant (ANOVA, *p*-value <0.0001) increase in serum levels of LDH, CEA, and AFP, with elevations of 3.29, 21.32, and 19.39-fold, respectively, compared to the control negative group. Furthermore, lung homogenate levels of MDA, TNF-α, and IL-1β were markedly (ANOVA, *p*-value <0.0001) elevated in the disease group, showing increases of 2.22, 5.79, and 6.94-fold, respectively. Conversely, the GSH level was reduced (ANOVA, *p*-value <0.0001) by 70.25 % in comparison to the control negative group.

The oral nintedanib group demonstrated a significant (ANOVA, *p*-value <0.0001) reduction in the levels of LDH, CEA, AFP, MDA, TNF-α, and IL-1β. The observed decreases were 42.88 %, 49.82 %, 56.55 %, 24.80 %, 18.79 %, and 30.50 %, respectively, compared to the disease group. Conversely, the level of GSH increased by 60.24 % in comparison to the disease group.

When compared to the disease group, the nebulized NLU50 group significantly (*p*-value <0.0001) reduced the levels of LDH, CEA, AFP, MDA, TNF-α, and IL-1β, with decreases of 55.74 %, 66.24 %, 69.87 %, 34.83 %, 51.91 %, and 48.76 %, respectively. Additionally, the GSH level increased by 120.5 %. In comparison to the oral nintedanib group, the nebulized NLU50 group also exhibited a notable (ANOVA, *p*-value <0.0001) decrease in levels of LDH, CEA, AFP, MDA, TNF-α, and IL-1β, with reductions of 22.51 %, 32.72 %, 30.73 %, 13.34 %, 40.78 %, and 26.27 %, respectively. Meanwhile, the GSH level rose by 37.5 %.

The nebulized NLU100 group significantly (*p*-value <0.0001) reduced the levels of LDH, CEA, AFP, MDA, TNF-α, and IL-1β compared to the disease group, with decreases of 65.42 %, 89.69 %, 94.33 %, 52.57 %, 78.26 %, and 81.10 %, respectively. Additionally, the GSH level increased (ANOVA, *p*-value <0.0001) by 200 % compared to the disease group. When comparing the nebulized NLU100 group to the oral nintedanib group, significant (ANOVA, p-value <0.0001) reductions were observed in the levels of LDH, CEA, AFP, MDA, TNF-α, and IL-1β, with decreases of 39.45 %, 79.45 %, 86.96 %, 36.93 %, 73.23 %, and 72.81 %, respectively. Additionally, the GSH level increased (ANOVA, *p*-value <0.0001) by 37.5 % in the nebulized NLU100 group compared to the oral nintedanib group. In comparison to the nebulized NLU50 group, the nebulized NLU100 group demonstrated significant (ANOVA, *p*-value <0.0001) reductions in the levels of LDH, CEA, AFP, MDA, TNF-α, and IL-1β by 21.87 %, 69.45 %, 81.19 %, 27.22 %, 54.79 %, and 63.12 %, respectively. Furthermore, the GSH level increased (ANOVA, *p*-value <0.0001) by 36.36 % compared to the nebulized NLU50.

The nebulized NLU200 group significantly (*p*-value <0.0001) reduced the levels of LDH, CEA, AFP, MDA, TNF-α, and IL-1β, with decreases of 66.10 %, 90.07 %, 94.46 %, 53.30 %, 78.40 %, and 81.43 %, respectively, compared to the disease group. Furthermore, the GSH level increased (ANOVA, *p*-value <0.0001) by 193.33 % in comparison to the disease group. The nebulized NLU200 group also displayed a notable (ANOVA, *p*-value <0.0001) decrease in levels of LDH, CEA, AFP, MDA, TNF-α, and IL-1β by 40.65 %, 80.21 %, 87.26 %, 37.89 %, 73.40 %, and 73.27 %, respectively, when compared to the oral nintedanib group. Furthermore, the GSH level increased (ANOVA, *p*-value <0.0001) by 83.33 % in comparison to the oral nintedanib group. The nebulized NLU200 group showed a significant (ANOVA, *p*-value <0.0001) difference in the levels of LDH, CEA, AFP, MDA, TNF-α, IL-1β, and GSH when compared to the nebulized NLU50 group. However, no significant (ANOVA, p-value >0.05) difference was found when comparing the nebulized NLU200 group to the nebulized NLU100 group.

### 3.8 Toxicity studies

**Fig. 6:**
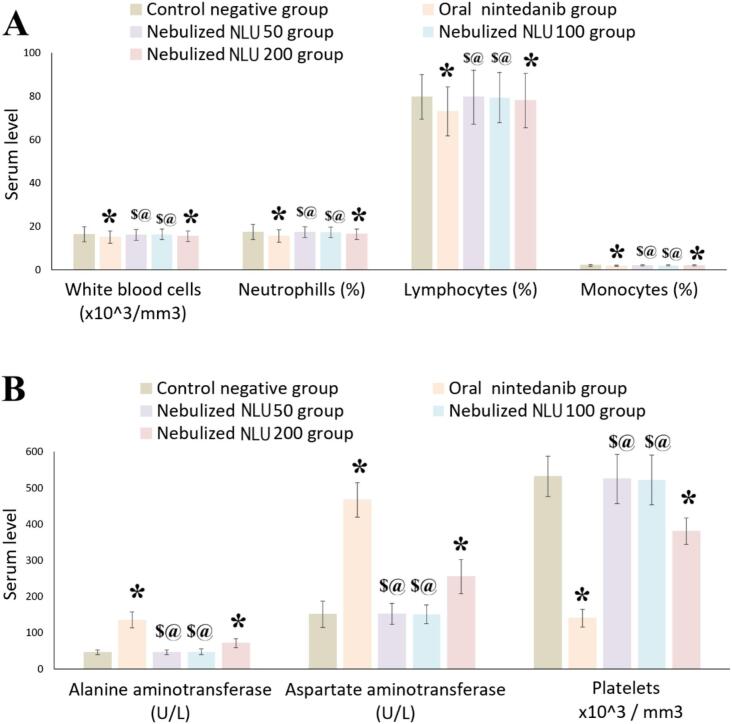
A) Effect of administration of oral nintedanib, nebulized nintedanib ufasomes at a dose of 50 mg/kg (NLU50), nebulized nintedanib ufasomes at a dose of 100 mg/kg (NLU100), and nebulized nintedanib ufasomes at a dose of 200 mg/kg (NLU200) on serum levels of immunity biomarkers; B) Effect of administration of oral nintedanib, nebulized nintedanib ufasomes at a dose of 50 mg/kg (NLU50), nebulized nintedanib ufasomes at a dose of 100 mg/kg (NLU100), and nebulized nintedanib ufasomes at a dose of 200 mg/kg (NLU200) on liver function and platelet counts. * indicates a significant difference compared to control negative group at p-value <0.05; $ indicates a significant difference compared to oral nintedanib group at p-value <0.05; @ indicates a significant difference compared to nebulized NLU200 group at p-value <0.05. The results were represented as mean ± SD (n = 6).

Fig. 6 illustrates the alterations in platelet count, immune response, and liver function resulting from various treatments.

The oral nintedanib group demonstrated a significant (ANOVA, *p*-value <0.0001) decrease in platelet count, as well as reductions in WBCs, neutrophils, lymphocytes, and monocytes, with decreases of 73.67 %, 7.97 %, 10.75 %, 8.41 %, and 9.01 %, respectively, compared to the control negative group. Furthermore, the levels of ALT and AST were markedly elevated (ANOVA, p-value <0.0001) in the oral nintedanib group, with increases of 2.98-fold and 3.09-fold, respectively, in comparison to the negative control group.

The nebulized NLU50 group demonstrated a significant (ANOVA, p-value <0.0001) increase in the counts of platelets, WBCs, neutrophils, lymphocytes, and monocytes, with increases of 3.75-fold, 107.33 %, 111.62 %, 109.04 %, and 108.65 %, respectively, when compared to the oral nintedanib group. Additionally, the nebulized NLU50 group significantly (p-value <0.0001) decreased the levels of ALT and AST by 65.93 % and 67.45 %, respectively, compared to the oral nintedanib group. No significant (ANOVA, p-value >0.05) differences were observed between the nebulized NLU50 group and the control group.

The nebulized NLU100 group significantly (ANOVA, p-value <0.0001) increased the counts of platelets, WBCs, neutrophils, lymphocytes, and monocytes, with increases of 3.73-fold, 108.88 %, 111.29 %, 108.63 %, and 108.10 %, respectively, when compared to the oral nintedanib group. Additionally, the nebulized NLU100 group significantly (p-value <0.0001) decreased the levels of ALT and AST by 65.18 % and 67.66 %, respectively, compared to the oral nintedanib group. No significant (ANOVA, *p*-value >0.05) differences between the nebulized NLU100 group and the control group. No significant (ANOVA, p-value >0.05) differences were observed between the nebulized NLU100 group and the nebulized NLU50 group.

The nebulized NLU200 group significantly (p-value <0.0001) decreased the platelet count, as well as reduced the levels of WBCs, neutrophils, lymphocytes, and monocytes, with decreases of 28.5 %, 4.91 %, 5.56 %, 2.13 %, and 3.11 %, respectively, compared to the control negative group. Furthermore, the levels of ALT and AST were markedly elevated (ANOVA, p-value <0.0001) in the nebulized NLU200 group, with increases of 56.62 % and 69.38 %, respectively, in comparison to the negative control group.

**Fig. 7:**
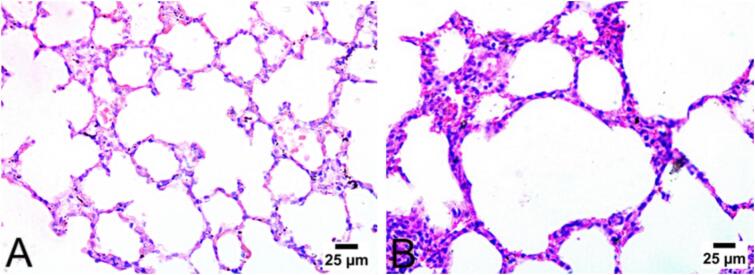
Histopathological (hematoxylin and eosin stain) observation of lung of A) control negative group; B) nebulized nintedanib ufasomes at a dose of 100 mg/kg (NLU100).

Fig. 7 presents the histopathological analysis of the nebulized NLU100 group. In the negative control group (Fig. 7A), the lung structures exhibited normal alveolar walls. The nebulized NLU100 group demonstrated no signs of malignancy, maintaining the normal structure of the bronchioles and alveolar walls.

## 4 Discussion

FFA, cholesterol, and NIS are necessary for ufasome synthesis (Abd-Elal et al., 2016; Gulshan et al., 2023; Illastria Rosalina et al., 2023). To reduce fluidity, pliability, and permeability, cholesterol plays a stabilizing role in vesicle membranes (Illastria Rosalina et al., 2023; Karal et al., 2022). The hydrophobic properties of Span 60, along with its specific phase transition temperature and unsaturated long alkyl chains, enabled it to achieve the highest EE% among all NIS vesicles tested (Abd-Elal et al., 2016; Illastria Rosalina et al., 2023). Among all FFA vesicles, oleic acid exhibits the lowest VS due to its very low melting point (Abd-Elal et al., 2016; Illastria Rosalina et al., 2023). Consequently, oleic acid, cholesterol, and Span 60 were selected. To determine the levels of oleic acid, cholesterol, and Span 60, we reviewed relevant literature and conducted preliminary investigations. The data indicate that formulations containing Span 60 in the range of 35 to 65 mg, cholesterol from 20 to 60 mg, and oleic acid from 10 to 50 mg achieved satisfactory EE% and VS. These results align with findings reported in other literature reviews (Abd-Elal et al., 2016; Gulshan et al., 2023; Illastria Rosalina et al., 2023; Albash et al., 2022).

The Box-Behnken design was employed to investigate the effects of Span 60, cholesterol, and oleic acid on the EE% and VS (Raghupathy and Amirthagadeswaran, 2014). This design is beneficial as it requires fewer experimental runs while yielding more reliable results (Raghupathy and Amirthagadeswaran, 2014). The Box-Behnken design evaluates multiple criteria to determine the optimal model for EE% and VS. A model is considered significant when the p-value is less than 0.05, accompanied by high F-statistic values, adequate precision, and a satisfactory R^2^. If the lack of fit is not statistically significant, the model is regarded as fitting well (Raghupathy and Amirthagadeswaran, 2014). Consequently, the quadratic model was selected. To confirm these findings, the design offers normal probability plots, where a normally distributed set of residuals is anticipated. Thus, the quadratic model proved effective in both predicting and validating the data for VS and EE%.

Cholesterol was shown to have a positive impact on both EE% and VS. The increased hydrophobicity and reduced bilayer permeability associated with higher cholesterol levels facilitated the formation of vesicles, which effectively encapsulated the hydrophobic drug. The findings reported by Ahmed et al. (Ahmed et al., 2022) support this observation. The NLU was able to increase its diameter by widening the distance between the ufasomal bilayers, a process enabled by the dense structure of cholesterol. As noted earlier, Aboud et al. (Aboud et al., 2024a) reported similar findings. Span 60 also positively influenced both EE% and VS, which can be attributed to its long-saturated alkyl chain. Aboud et al. (Aboud et al., 2024a) and Albash et al. (Albash et al., 2022) reported similar findings. Furthermore, oleic acid positively affected both EE% and VS due to the hydrophobic properties of its long alkyl chain (C18) and its attractive steric forces. These results align with the findings of Gabr et al. (Gabr et al., 2017) and Pinilla et al. (Pinilla et al., 2020).

The numerical strategy focused on identifying the optimized NLU. After exploring various options, the program settled on the formulation that had the highest DI. To confirm these findings, the optimal NLU formulation was subjected to tests measuring VS and EE% in triplicate. The design effectively identified the optimal NLU formulation, demonstrated by an error margin of less than 1 % between the actual data and the predicted data generated by the program. To enhance the drug's bioavailability, Aboud et al. developed nintedanib-loaded ufasomes (Aboud et al., 2024a). Although they reported positive outcomes concerning VS and EE%, the current study introduces better results. The optimized NLU resulted in a 1.42-fold increase in EE% and a 24.99 % reduction in VS compared to the ufasomes.

The PDI is a numerical metric used to estimate the uniformity of nanovesicle sizes (Abd-Elal et al., 2016). The size distribution is homogeneous and uniform when the PDI is low. However, it becomes polydisperse and heterogeneous at high PDI values. The optimized NLU, characterized by a low PDI value, signifies that the vesicles are consistently and uniformly distributed. The stability of these vesicles can be indirectly assessed using ZP (Fatima et al., 2022). The negative ZP value of the optimized NLU results from oleic acid's presence, which contributes additional negative charges to molecules upon ionization due to its carboxylic group. DSC was employed to investigate the physical states and interactions of the pharmaceutical components (Abd-Elal et al., 2016). The lack of peaks in the optimized NLU thermograph suggests that nintedanib exists in an amorphous state. The FTIR spectra of the optimized NLU are similar to those of its components, suggesting compatibility among them.

To ensure constant sink conditions for the release study, solubility equilibrium studies were conducted (Abouelmagd et al., 2015). A volume of 50 ml of PT80 was selected as the release medium because it exceeded the saturated solubility of nintedanib. This result lends credence to the report of Liu et al. (Liu et al., 2018). The sustainability of the optimized NLU resulted from the synergistic hydrophobic effects of oleic acid and Span 60. Compared to free nintedanib, the optimal NLU formulation exhibited improved MDT performance and a more favorable sustained release pattern. Aboud et al. (Aboud et al., 2024a) and Aboud et al. (Aboud et al., 2024b) reached similar conclusions. However, the results of this investigation were more favorable. In comparison to the optimal ufasomes (Aboud et al., 2024a) and optimal micelles (Aboud et al., 2024b), our optimized NLU improved nintedanib's sustainability by a factor of 1.36 and 1.16, respectively.

The drug release kinetics and mechanism were analyzed using the DDsolver program (Kirimlioğlu and Öztürk, 2020). This program identifies the best-fitting model for the data by considering R^2^, AIC, and MSC. The selected model outperformed the alternatives, exhibiting impressive R^2^ and MSC values alongside a low AIC. Consequently, predictions derived from the Korsmeyer-Peppas model were the most accurate. To assess the mechanism of drug release, the release exponent “n” from the Korsmeyer-Peppas model was applied (Kirimlioğlu and Öztürk, 2020; Abdul Rasool et al., 2021). The findings of the current study demonstrated Fickian diffusion because the value of n is less than 0.45.

The optimized NLU was stable after 90 days at temperatures of 4, 25, and 40 °C. The higher negative charge of oleic acid may explain this outcome, as it likely contributed to the reduced extent of ufasome coalescence.

The aerodynamic performance of the optimized NLU was evaluated using the ACI, focusing on FPD, FPF, MMAD, and GSD. The size and distribution of aerosolized particles influence both the quantity of deposited medication and the specific locations where it is deposited. Enhanced aerosolization performance occurs when FPD and FPF values are elevated, while MMAD and GSD values are minimized (Rahimkhani et al., 2017; Elkomy et al., 2022). The increase in FPD and FPF is promising, as it suggests that nintedanib has a higher likelihood of reaching its intended target in the lower airways, where it can exert its maximum effect (Rahimkhani et al., 2017; Elkomy et al., 2022). A low MMAD indicates a greater likelihood of deep deposition in lung tissue (Rahimkhani et al., 2017; Elkomy et al., 2022). Additionally, when the GSD is low, it allows for reliable prediction and replication of therapeutic outcomes, as this reflects a narrower size distribution (Rahimkhani et al., 2017; Elkomy et al., 2022). The optimal NLU formulation exhibited superior aerodynamic characteristics compared to the nintedanib, specifically regarding FPD, FPF, MMAD, and GSD. The results suggest that the optimized NLU is both inhalable and respirable, facilitating a more accurate accumulation of nintedanib in the lungs.

Aboud et al. (Aboud et al., 2024a) and Shukla et al. (Shukla et al., 2022) reached similar conclusions. However, the results of this investigation were more favorable. Our optimized NLU achieved a 10.29 % reduction in MMAD and a 1.05-fold increase in FPF, surpassing the performance of optimal ufasomes (Aboud et al., 2024a) in terms of lung deposition potential. Our optimized NLU achieved 1.08-fold, 35.58 %, and 21.5 % increases in FPF, MMAD, and GSD, surpassing the performance of optimal niosomes (Shukla et al., 2022) in terms of lung deposition potential.

The A549 was utilized to investigate the cytotoxicity of the optimal NLU formulation. The findings suggest that this formulation exhibits greater cytotoxicity against the cancer cells, as evidenced by a notable decrease in cell viability compared to the nintedanib suspension. Additionally, the optimal NLU formulation demonstrates superior performance over the nintedanib suspension at lower concentrations, as indicated by its low IC50 value. These results imply that the optimized NLU might outperform the nintedanib suspension in terms of reducing tumor cell proliferation. The safety profile of the optimal NLU formulation was evaluated using BEAS-2B normal cells, and the results show that this formulation is both safe and biocompatible.

According to our findings, Kala et al. (Kala and Chinni, 2022) and Shukla et al. (Shukla et al., 2022) are in agreement; however, the results of this investigation were superior. The optimized NLU enhanced nintedanib cytotoxicity by factors of 1.69 and 1.28, respectively, compared to optimal liposomes (Kala and Chinni, 2022) and optimal niosomes (Shukla et al., 2022).

The relative bioavailability and sustainability of the optimized NLU were greater than that of free nintedanib, as indicated by the pharmacokinetic study of the drug. The incorporation of Span 60 and oleic acid enhanced the solubility of nintedanib, leading to improved absorption. Our results align with those of Kala et al. (Kala and Chinni, 2022), Zhu et al. (Zhu et al., 2020), Aboud et al. (Aboud et al., 2024a), Aboud et al. (Aboud et al., 2024b), and Kaur et al. (Kaur et al., 2024). However, the findings of this study were superior. The optimized NLU enhanced nintedanib bioavailability by factors of 1.15, 2, 1.16, 1.72, and 2.26, respectively, compared to optimal liposomes (Kala and Chinni, 2022), optimal nanostructured lipid carriers (Zhu et al., 2020), optimal ufasomes (Aboud et al., 2024a), optimal micelles (Aboud et al., 2024b), and optimal solid lipid nanoparticles (Kaur et al., 2024).

CT scans confirmed these findings. The nebulized NLU group exhibited that most of the drug was concentrated in the lungs, suggesting it was specifically targeted to this organ. Consequently, the optimal NLU may enhance nintedanib's targeted capability by serving as a more efficient drug carrier.

Lewis lung carcinoma cells have played a crucial role in advancing fundamental discoveries in cancer biology and translational medicine (Tanaka et al., 2023; Xu et al., 2022; He et al., 2024). They serve as an effective model for studying the underlying pathology of lung cancer and for developing novel treatments. To ensure the successful induction of lung cancer and evaluate the anti-tumor effectiveness of each treatment, various parameters were monitored, including tumor indicators and histological features. The roles of LDH, AFP, and CEA in the progression, invasion, and metastasis of lung cancer have been extensively studied and documented (Hamzawy et al., 2017; Zhou et al., 2014; Willder et al., 2012). A notable increase in MDA levels and a decrease in GSH activity indicated changes in lipid peroxidation and oxidative stress. Inflammatory cytokines such as TNF-α and IL-1β significantly affect lung adenocarcinoma. Inflammatory cytokines, lipid peroxidation, oxidative stress, and tumor biomarkers were all significantly elevated in the disease group compared to the negative control group. The findings of this study confirm that LLC is a potent carcinogen for lung cancer, effectively inducing the disease.

Oral nintedanib treatment significantly decreased tumor biomarkers, lipid peroxidation, and inflammatory cytokines compared to the disease group. Nintedanib exhibits a wide range of anti-tumor, anti-inflammatory, and anti-oxidative effects. It may exert an antioxidative influence by reducing levels of reactive oxygen species and transglutaminase-2 (Boxhammer et al., 2020). Additionally, nintedanib lowers the expression of chemokines and cytokines while inhibiting macrophage infiltration (Wang and Qu, 2022). In conjunction with promoting apoptosis and modulating proinflammatory factor expression, nintedanib also inhibits hyperproliferation (Li et al., 2021). The histopathological study confirms nintedanib's antiproliferative properties. The results of this study are consistent with the findings of Wang et al. and Hao et al. (Wang and Qu, 2022; Hao et al., 2025). Moreover, nebulized administration of NLU at various doses demonstrated greater anti-tumor, anti-inflammatory, and anti-oxidative benefits compared to oral nintedanib. Improved efficacy results from the various advantages of sustained release, such as enhanced bioavailability, increased accumulation in tumor tissue, prolonged plasma circulation, and greater drug exposure to the tumor. The histopathological study further confirms the enhanced antiproliferative properties of nintedanib.

Changes in platelet count, immune function, and liver enzyme levels indicate the toxicity profile of nintedanib. Mice administered oral nintedanib exhibited elevated liver enzymes, thrombocytopenia, and reduced immune function. The reduction in platelet counts and immune response may be linked to the drug's mechanism of action, which includes inhibiting PDGF receptor (Liu et al., 2021; Dumic et al., 2023). Additionally, as an inhibitor of tyrosine kinase, nintedanib therapy is associated with increased liver enzyme levels (Raschi et al., 2022). These findings are consistent with those reported by Yan et al. and Bronte et al. (Yan et al., 2023; Bronte et al., 2016). Nebulized NLU restored levels of platelet count, white blood cells, neutrophils, lymphocytes, monocytes, ALT, and AST when compared to oral nintedanib. This improvement is believed to stem from increased tumor exposure to the drug.

The efficacy and toxicity of nebulized NLU were evaluated at various doses. The results indicated that the efficacy of nebulized NLU at doses of 100 mg/kg and 200 mg/kg was superior to that of 50 mg/kg; however, toxicity was observed at the 200 mg/kg dose. No significant difference in efficacy was found between the 100 mg/kg and 200 mg/kg doses. This suggests that nebulized NLU at a dose of 100 mg/kg could serve as an effective therapy for NSCLC. Nebulized NLU at a dose of 100 mg/kg has the potential to enhance patient compliance and minimize side effects associated with nintedanib by decreasing both the dosage and frequency of administration. The formulation facilitates localized pulmonary drug delivery. Consequently, the drug quickly arrives at its site of action, avoiding first-pass metabolism in the liver and leading to a rapid onset of therapeutic effects. Consequently, the nintedanib dosage can be decreased, which helps minimize side effects. Patients may be more compliant and experience fewer side effects due to NLU's sustained release effect, which distributes the dose evenly throughout the lungs. This formulation may also reduce the frequency of dosing. The nebulized NLU demonstrates superior bioavailability, enabling a lower dosage to achieve equivalent or even enhanced therapeutic effects.

## 5 Conclusion

The optimized NLU enhances the sustainability and bioavailability of nintedanib. It is respirable and, when nebulized, facilitates a more targeted accumulation of nintedanib in the lungs. The optimized NLU was found to be safe in BEAS-2B normal lung cells but cytotoxic in A549 lung cell lines. This formulation shows greater potential than nintedanib for reducing tumor cell proliferation. The nebulized NLU restored the counts of platelets, neutrophils, lymphocytes, and monocytes, as well as the levels of ALT and AST, compared to oral nintedanib. The efficacy and toxicity of the nebulized NLU were evaluated at various doses. A nebulized NLU at a dose of 100 mg/kg may offer efficient and safe therapy for NSCLC.

## CRediT authorship contribution statement

**Salman M. Ghazwani:** Writing – review & editing, Software, Resources, Formal analysis, Conceptualization. **Sami Alhazmi:** Writing – review & editing, Writing – original draft, Software, Methodology, Conceptualization. **Salhah M. Ghazwani:** Writing – review & editing, Resources, Project administration, Methodology, Funding acquisition, Data curation, Conceptualization. **Hussam M. Shubaily:** Writing – review & editing, Validation, Software, Methodology, Conceptualization. **Ahmed M. Wafi:** Writing – review & editing, Writing – original draft, Methodology, Conceptualization. **Naifa Alenazi:** Writing – original draft, Software, Methodology, Data curation. **Marwa Qadri:** Writing – review & editing, Writing – original draft, Software, Methodology, Data curation. **Amal Naif Alshammari:** Writing – review & editing, Writing – original draft, Methodology, Investigation, Formal analysis, Conceptualization. **Wedad Mawkili:** Writing – original draft, Software, Methodology, Conceptualization. **Jobran M. Moshi:** Writing – review & editing, Writing – original draft, Software, Conceptualization. **Zenat Khired:** Writing – original draft, Software, Conceptualization. **Salama A. Salama:** Writing – review & editing, Writing – original draft, Software, Methodology.

## Consent for publication

Not applicable.

## Ethical approval and consent to participate

The animal approval was conducted in accordance with the ethical standards set forth by the ARRIVE guidelines. The Institutional Animal Ethics Committee (IACUC024–013) reviewed and approved the animal studies conducted under the research.

## Funding

Princess Nourah bint Abdulrahman University Researchers Supporting Project number (PNURSP2025R891), 10.13039/501100004242Princess Nourah bint Abdulrahman University, Riyadh, Saudi Arabia

## Declaration of competing interest

The authors declare that they have no known competing financial interests or personal relationships that could have appeared to influence the work reported in this paper.

## Data Availability

Data will be made available on request.
